# 2,5-Bis(5-bromo-2-thien­yl)thio­phene

**DOI:** 10.1107/S1600536809030864

**Published:** 2009-08-08

**Authors:** Mamoun M. Bader

**Affiliations:** a Department of Chemistry, Pennsylvania State University at Hazleton, 76 University Drive, Hazleton, PA 18202, USA

## Abstract

In the crystal structure of the title compound, C_12_H_6_Br_2_S_3_, the mol­ecules are planar (r.m.s. deviation = 0.06 Å). Consecutive mol­ecules do not stack in a planar fashion. There is an angle of 81.7 (12)° between the planes of the closest mol­ecules.

## Related literature

For related structures, see: Pyrka *et al.* (1988 ▶). For literature related to synthesis, see: Hoffmann & Carlsen (1999 ▶); Mei *et al.* (2009 ▶). For a recent review of oligothio­phenes, see: Mishra *et al.* (2009 ▶).
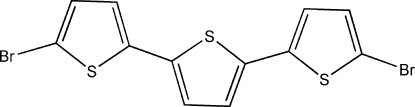


## Experimental

### 

#### Crystal data

C_12_H_6_Br_2_S_3_
*M* *_r_* = 406.17
Orthorhombic, 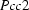

*a* = 7.6216 (16) Å
*b* = 30.003 (6) Å
*c* = 5.8841 (13) Å
*V* = 1345.5 (5) Å^3^
*Z* = 4
Mo *K*α radiation
μ = 6.46 mm^−1^
*T* = 173 K
0.37 × 0.24 × 0.10 mm

#### Data collection

Siemens SMART Platform CCD diffractometer
Absorption correction: multi-scan (*SADABS*; Sheldrick, 2008*a* ▶) *T*_min_ = 0.184, *T*_max_ = 0.524
9565 measured reflections
3045 independent reflections
2818 reflections with *I* > 2σ(*I*)
*R*_int_ = 0.035

#### Refinement

*R*[*F* ^2^ > 2σ(*F* ^2^)] = 0.053
*wR*(*F* ^2^) = 0.114
*S* = 1.25
3045 reflections
155 parameters
1 restraint
H-atom parameters constrained
Δρ_max_ = 1.24 e Å^−3^
Δρ_min_ = −0.62 e Å^−3^
Absolute structure: Flack (1983 ▶), 1341 Friedel pairs
Flack parameter: 0.00 (7)

### 

Data collection: *SMART* (Bruker, 2001 ▶); cell refinement: *SAINT* (Bruker, 2001 ▶); data reduction: *SAINT*; program(s) used to solve structure: *SHELXS97* (Sheldrick, 2008*b* ▶); program(s) used to refine structure: *SHELXL97* (Sheldrick, 2008*b* ▶); molecular graphics: *SHELXTL* (Sheldrick, 2008*b* ▶); software used to prepare material for publication: *SHELXTL*.

## Supplementary Material

Crystal structure: contains datablocks I, global. DOI: 10.1107/S1600536809030864/ng2619sup1.cif

Structure factors: contains datablocks I. DOI: 10.1107/S1600536809030864/ng2619Isup2.hkl

Additional supplementary materials:  crystallographic information; 3D view; checkCIF report

## Figures and Tables

**Fig. 1.**
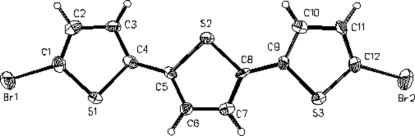
2,5-bis(5-bromothiophen-2-yl)thiophene.

**Fig. 2.**
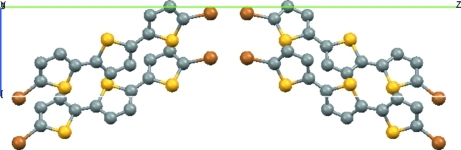
Crystal packing viewed along the a axis.

## supplementary crystallographic information

### Comment

Dibromothiophenes are important building blocks in materials chemistry. They are mainly used in the prepartaion of various thiophene oligomers and polymers utilizing coupling reactions such as Stille and Suzuki couplings.

For literature related to the synthesis see: Hoffman & Carlsen (1999) and Mei (2009). For a recent review on synthesis and applications of oligothiophenes,see: Mishra (2009).

### Experimental

Synthesis was carried out following literature procedures (Hoffman) as follows: to a a solution of terthiophene dissolved in chloroform was added 2 equivalents of *N*-bromosuccinimde and the reaction mixture was stirred at room temperature for 2 h. The reaction mixture was then extracted with water and product obtained by evaporation of chloroform and recrystallized twice from hexanes. The crystals were very thin, hence the large number in the second weighting scheme.

### Refinement

The structure was solved using *SHELXS97* and refined using *SHELXL97* (Sheldrick, 2008). The space group Pcc2 was determined based on systematic absences and intensity statistics. A direct-methods solution was calculated which provided most non-hydrogen atoms from the E-map. Full-matrix least squares / difference Fourier cycles were performed which located the remaining non-hydrogen atoms. All non-hydrogen atoms were refined with anisotropic displacement parameters. All hydrogen atoms were placed in ideal positions and refined as riding atoms with relative isotropic displacement parameters. The final full matrix least squares refinement converged to R1 = 0.0527 and wR2 = 0.1169 (F2, all data).

### Crystal data

**Table:**

C_12_H_6_Br_2_S_3_	*F*(000) = 784
*M**_r_* = 406.17	*D*_x_ = 2.005 Mg m^−^^3^
Orthorhombic, *P**c**c*2	Mo *K*α radiation, λ = 0.71073 Å
Hall symbol: P 2 -2c	Cell parameters from 903 reflections
*a* = 7.6216 (16) Å	θ = 2.7–27.5°
*b* = 30.003 (6) Å	µ = 6.46 mm^−^^1^
*c* = 5.8841 (13) Å	*T* = 173 K
*V* = 1345.5 (5) Å^3^	Plate, pale yellow
*Z* = 4	0.37 × 0.24 × 0.10 mm

### Data collection

**Table:**

Siemens SMART Platform CCD diffractometer	3045 independent reflections
Radiation source: fine-focus sealed tube	2818 reflections with *I* > 2σ(*I*)
graphite	*R*_int_ = 0.035
ω scans	θ_max_ = 27.5°, θ_min_ = 2.7°
Absorption correction: multi-scan (*SADABS*; Sheldrick, 2008a)	*h* = −9→9
*T*_min_ = 0.184, *T*_max_ = 0.524	*k* = −38→38
9565 measured reflections	*l* = −7→7

### Refinement

**Table:**

Refinement on *F*^2^	Secondary atom site location: difference Fourier map
Least-squares matrix: full	Hydrogen site location: inferred from neighbouring sites
*R*[*F*^2^ > 2σ(*F*^2^)] = 0.053	H-atom parameters constrained
*wR*(*F*^2^) = 0.114	*w* = 1/[σ^2^(*F*_o_^2^) + (0.0403*P*)^2^ + 2.9087*P*] where *P* = (*F*_o_^2^ + 2*F*_c_^2^)/3
*S* = 1.25	(Δ/σ)_max_ = 0.001
3045 reflections	Δρ_max_ = 1.24 e Å^−^^3^
155 parameters	Δρ_min_ = −0.62 e Å^−^^3^
1 restraint	Absolute structure: Flack (1983), 1341 Friedel pairs
Primary atom site location: structure-invariant direct methods	Flack parameter: 0.00 (7)

### Special details

**Table:**

Geometry. All e.s.d.'s (except the e.s.d. in the dihedral angle between two l.s. planes)are estimated using the full covariance matrix. The cell e.s.d.'s are takeninto account individually in the estimation of e.s.d.'s in distances, anglesand torsion angles; correlations between e.s.d.'s in cell parameters are onlyused when they are defined by crystal symmetry. An approximate (isotropic)treatment of cell e.s.d.'s is used for estimating e.s.d.'s involving l.s. planes.
Refinement. Refinement of *F*^2^ against ALL reflections. The weighted *R*-factor *wR* and goodness of fit *S* are based on *F*^2^, conventional *R*-factors *R* are based on *F*, with *F* set to zero for negative *F*^2^. The threshold expression of *F*^2^ > σ(*F*^2^) is used only for calculating *R*-factors(gt) *etc*. and is not relevant to the choice of reflections for refinement. *R*-factors based on *F*^2^ are statistically about twice as large as those based on *F*, and *R*- factors based on ALL data will be even larger. The structure refined as a merohedral inversion twin, whose mass ratio converged to 61:39.

### Fractional atomic coordinates and isotropic or equivalent isotropic displacement parameters (Å^2^)

**Table:**

	*x*	*y*	*z*	*U*_iso_*/*U*_eq_	
Br1	0.69877 (9)	0.96042 (2)	1.02236 (11)	0.0374 (2)	
Br2	0.71126 (13)	0.54014 (2)	0.07093 (13)	0.0532 (3)	
S1	0.6713 (2)	0.86007 (5)	0.9031 (3)	0.0271 (3)	
S2	0.81817 (19)	0.76622 (5)	0.3646 (3)	0.0232 (3)	
S3	0.6749 (2)	0.62433 (5)	0.3757 (3)	0.0279 (3)	
C1	0.7418 (8)	0.9124 (2)	0.8288 (11)	0.0253 (13)	
C2	0.8278 (8)	0.9124 (2)	0.6263 (12)	0.0287 (14)	
H2	0.8761	0.9384	0.5584	0.034*	
C3	0.8376 (7)	0.86976 (19)	0.5284 (11)	0.0238 (12)	
H3	0.8933	0.8642	0.3868	0.029*	
C4	0.7597 (7)	0.8370 (2)	0.6553 (10)	0.0215 (12)	
C5	0.7360 (7)	0.7908 (2)	0.6122 (10)	0.0172 (12)	
C6	0.6539 (7)	0.75937 (18)	0.7416 (10)	0.0197 (12)	
H6	0.6009	0.7660	0.8838	0.024*	
C7	0.6546 (7)	0.71661 (19)	0.6471 (10)	0.0201 (12)	
H7	0.6016	0.6916	0.7182	0.024*	
C8	0.7397 (8)	0.71439 (19)	0.4407 (12)	0.0190 (12)	
C9	0.7621 (7)	0.67576 (19)	0.2972 (10)	0.0175 (11)	
C10	0.8467 (8)	0.6726 (2)	0.0932 (10)	0.0233 (12)	
H10	0.9036	0.6972	0.0231	0.028*	
C11	0.8421 (8)	0.62914 (19)	−0.0055 (11)	0.0249 (12)	
H11	0.8927	0.6216	−0.1479	0.030*	
C12	0.7553 (9)	0.5999 (2)	0.1323 (12)	0.0269 (13)	

### Atomic displacement parameters (Å^2^)

**Table:**

	*U*^11^	*U*^22^	*U*^33^	*U*^12^	*U*^13^	*U*^23^
Br1	0.0447 (4)	0.0317 (4)	0.0359 (4)	0.0053 (3)	0.0051 (4)	−0.0089 (3)
Br2	0.0814 (7)	0.0272 (4)	0.0512 (7)	−0.0077 (4)	0.0045 (5)	−0.0099 (4)
S1	0.0315 (8)	0.0282 (7)	0.0217 (8)	−0.0025 (6)	0.0079 (6)	−0.0021 (6)
S2	0.0252 (7)	0.0259 (7)	0.0185 (7)	−0.0031 (6)	0.0045 (6)	0.0006 (6)
S3	0.0338 (8)	0.0234 (7)	0.0263 (8)	−0.0046 (6)	0.0066 (7)	0.0012 (7)
C1	0.028 (3)	0.025 (3)	0.023 (3)	0.002 (2)	−0.005 (3)	−0.003 (3)
C2	0.023 (3)	0.029 (3)	0.035 (3)	−0.002 (2)	−0.003 (3)	0.009 (3)
C3	0.026 (3)	0.024 (3)	0.021 (3)	0.000 (2)	0.006 (3)	0.004 (3)
C4	0.018 (3)	0.030 (3)	0.017 (3)	0.003 (2)	0.000 (2)	0.000 (2)
C5	0.012 (3)	0.026 (3)	0.013 (3)	−0.001 (2)	−0.003 (2)	−0.004 (2)
C6	0.019 (3)	0.023 (3)	0.017 (3)	−0.001 (2)	−0.001 (2)	0.001 (2)
C7	0.012 (3)	0.026 (3)	0.022 (3)	0.000 (2)	−0.001 (2)	0.005 (2)
C8	0.014 (2)	0.017 (3)	0.026 (3)	−0.002 (2)	0.004 (2)	0.010 (2)
C9	0.016 (3)	0.015 (3)	0.021 (3)	0.002 (2)	−0.001 (2)	0.000 (2)
C10	0.020 (3)	0.029 (3)	0.021 (3)	0.002 (2)	−0.001 (2)	0.003 (2)
C11	0.028 (3)	0.023 (3)	0.024 (3)	0.005 (2)	0.004 (3)	−0.006 (2)
C12	0.035 (3)	0.022 (3)	0.024 (3)	−0.004 (3)	0.000 (3)	−0.006 (3)

### Geometric parameters (Å, °)

**Table:**

Br1—C1	1.864 (6)	C4—C5	1.419 (8)
Br2—C12	1.858 (6)	C5—C6	1.365 (8)
S1—C1	1.717 (7)	C6—C7	1.398 (8)
S1—C4	1.749 (6)	C6—H6	0.9500
S2—C8	1.725 (6)	C7—C8	1.379 (9)
S2—C5	1.750 (6)	C7—H7	0.9500
S3—C12	1.722 (7)	C8—C9	1.444 (8)
S3—C9	1.742 (6)	C9—C10	1.366 (8)
C1—C2	1.360 (10)	C10—C11	1.428 (8)
C2—C3	1.404 (9)	C10—H10	0.9500
C2—H2	0.9500	C11—C12	1.367 (9)
C3—C4	1.370 (8)	C11—H11	0.9500
C3—H3	0.9500		
			
C1—S1—C4	91.7 (3)	C7—C6—H6	122.9
C8—S2—C5	92.3 (3)	C8—C7—C6	113.4 (5)
C12—S3—C9	91.2 (3)	C8—C7—H7	123.3
C2—C1—S1	111.9 (5)	C6—C7—H7	123.3
C2—C1—Br1	128.4 (5)	C7—C8—C9	127.6 (5)
S1—C1—Br1	119.8 (4)	C7—C8—S2	110.4 (5)
C1—C2—C3	112.7 (6)	C9—C8—S2	122.1 (5)
C1—C2—H2	123.6	C10—C9—C8	128.7 (5)
C3—C2—H2	123.6	C10—C9—S3	110.6 (4)
C4—C3—C2	114.0 (6)	C8—C9—S3	120.7 (4)
C4—C3—H3	123.0	C9—C10—C11	114.2 (6)
C2—C3—H3	123.0	C9—C10—H10	122.9
C3—C4—C5	131.2 (5)	C11—C10—H10	122.9
C3—C4—S1	109.7 (5)	C12—C11—C10	110.9 (5)
C5—C4—S1	119.1 (4)	C12—C11—H11	124.5
C6—C5—C4	129.3 (5)	C10—C11—H11	124.5
C6—C5—S2	109.7 (4)	C11—C12—S3	113.0 (5)
C4—C5—S2	121.0 (4)	C11—C12—Br2	126.3 (5)
C5—C6—C7	114.3 (5)	S3—C12—Br2	120.6 (4)
C5—C6—H6	122.9		
			
C4—S1—C1—C2	0.0 (5)	C6—C7—C8—C9	−179.1 (6)
C4—S1—C1—Br1	−179.1 (4)	C6—C7—C8—S2	−0.3 (6)
S1—C1—C2—C3	0.1 (7)	C5—S2—C8—C7	0.0 (5)
Br1—C1—C2—C3	179.1 (5)	C5—S2—C8—C9	179.0 (5)
C1—C2—C3—C4	−0.2 (8)	C7—C8—C9—C10	−179.5 (6)
C2—C3—C4—C5	177.2 (6)	S2—C8—C9—C10	1.8 (9)
C2—C3—C4—S1	0.2 (7)	C7—C8—C9—S3	0.8 (9)
C1—S1—C4—C3	−0.1 (5)	S2—C8—C9—S3	−178.0 (3)
C1—S1—C4—C5	−177.6 (5)	C12—S3—C9—C10	0.2 (5)
C3—C4—C5—C6	−178.4 (7)	C12—S3—C9—C8	180.0 (5)
S1—C4—C5—C6	−1.6 (9)	C8—C9—C10—C11	−179.2 (6)
C3—C4—C5—S2	2.0 (9)	S3—C9—C10—C11	0.6 (7)
S1—C4—C5—S2	178.8 (3)	C9—C10—C11—C12	−1.3 (8)
C8—S2—C5—C6	0.2 (5)	C10—C11—C12—S3	1.4 (7)
C8—S2—C5—C4	179.8 (5)	C10—C11—C12—Br2	178.2 (5)
C4—C5—C6—C7	−180.0 (5)	C9—S3—C12—C11	−1.0 (5)
S2—C5—C6—C7	−0.4 (7)	C9—S3—C12—Br2	−177.9 (4)
C5—C6—C7—C8	0.4 (7)		
